# Quadratus lumborum block for postoperative analgesia after cesarean section: a meta-analysis of randomized controlled trials with trial sequential analysis

**DOI:** 10.1038/s41598-021-96546-7

**Published:** 2021-09-13

**Authors:** Zhigang Zhao, Kaiming Xu, Yanting Zhang, Gang Chen, Youfa Zhou

**Affiliations:** 1 Department of Anesthesiology, Shaoxing Campus, Sir Run Run Shaw Hospital, School of Medicine, Zhejiang University, Shaoxing, China; 2 Department of Anesthesiology, Shaoxing Shangyu Second People’s Hospital, Shaoxing, China; 3 Department of Anesthesiology, Sir Run Run Shaw Hospital, School of Medicine, Zhejiang University, Qingchun East Road No. 3, Hangzhou, 310020 China

**Keywords:** Outcomes research, Clinical trial design

## Abstract

The aim of this study was to assess the analgesic efficacy of QLB versus controls in women undergoing cesarean section (CS). We systematically searched Cochrane Library, PUBMED, EMBASE, VIP, WANFANG, and China National Knowledge Infrastructure. Trials were eligible if parturients received QLB during CS. GRADE system was used to assess the certainty of evidence and Trial sequential analyses (TSA) were performed to determine whether the results are supported by sufficient data. Thirteen studies involving 1269 patients were included. Compared to controls, QLB significantly reduced the cumulative postoperative intravenous opioid consumption (in milligram morphine equivalents) at 24 h (MD, − 11.51 mg; 95% CI − 17.05 to − 5.96) and 48 h (MD, − 15.87 mg; 95% CI − 26.36 to − 5.38), supported by sufficient data confirmed by TSA. The postoperative pain scores were significantly reduced by QLB at 4 h, 6 h, 12 h, 24 h, and 48 h postoperatively by QLB compared with control. Moreover, the time to first request for rescue analgesic and the incidence of PONV were also significantly reduced by QLB. The quality of evidence of most results were low and moderate assessed by GRADE.

## Introduction

Cesarean section (CS) is one of the most common surgical procedures performed in gynecology and obstetrics in the world, which is in a steadily increasing trend^1^. Effective management of postoperative pain is vital to allow the newly delivered mothers to care for their newborn infants^2,3^. Moreover, effective postoperative analgesia help the parturients for early ambulation which may reduce the risk of thromboembolism and development of chronic pain^4^. Opioids are still considered as the cornerstone of the postoperative analgesia, while they are associated with significant adverse effects such as nausea, vomiting, and pruritis which may reduce the overall patient satisfaction^5^. Moreover, the risk of potential for opioid misuse and delayed maternal respiratory depression make need to identify opioid-sparing techniques^6^.

Quadratus lumborum block (QLB) has gained increasing attention of researches for its potential capability to provide both visceral and somatosensory pain relief^7^. It was reported that this effect was probably due to the wider spread of the local anesthetic beyond the transversus abdominis plane into the paravertebral space^8^. There are different types of QLB according to the position of the needle tip and the approach of the needle. An increasing number of studies have showed that QLB can reduce postoperative pain and morphine requirements after CS when compared with sham block or placebo^9–18^. However, some trials have yielded conflicting results that QLB did not reduce postoperative morphine consumption and pain scores^19,20^. A recent meta-analysis evaluated the analgesic effectiveness of QLB in cesarean delivery with and without spinal morphine and showed that the block can improves post-cesarean analgesia only in the absence of spinal morphine^21^. However, there was no subgroup analysis according to different types of QLB in the above meta-analysis. Moreover, it is very necessary for us to conduct trial sequential analysis (TSA) to reduce the risk of a type I error when a meta-analysis includes a small number of studies or the sample size is not large enough^22,23^.

Therefore, we conducted this meta-analysis with TSA of randomized controlled trials to identify the potential clinical role of QLB after CS.

## Materials and methods

We prepared this manuscript according to the Preferred Reporting Items for Systematic Reviews and Meta-Analyses (PRISMA).

### Eligibility criteria

Randomized controlled studies that allocated pregnant women undergoing cesarean section to receive QLB were considered for inclusion. We accepted all variations of the QLB technique. Trials were excluded if QLB was performed in conjunction with other blocks. Eligible comparators included systemic analgesia alone (i.e., no block or sham block, as Control). No language limitation were adopted on study inclusion; any non-English studies were translated by an online translator.

### Search strategy

A systematic search strategy was conducted in the Cochrane Library, PUBMED, EMBASE, VIP, WANFANG, and China National Knowledge Infrastructure (CNKI). These databases were searched from inceptions to April 25, 2020 without language limitation. The search strategy included the following terms: (cesarean OR cesarean section OR caesarean OR c-section OR "abdominal delivery") AND ((quadratus lumborum OR (abdominal muscles [mesh] AND nerve block[mesh]))). Moreover, we also searched reference lists of included articles for any relevant trials. Data from conference proceedings and abstracts were not considered if they were not published as full articles.

### Study selection

The process of study selection was consistent with the description in our previous study^24^. Retrieved studies were imported into Endnote (version X7; Thomson Reuters), where duplications were detected and deleted automatically. Two independent reviewers initially scanned the titles and abstract of retrieved studies according to the established eligibility criteria to exclude the obvious irrelevant studies. The full-text of potentially eligible articles were then retrieved and assessed again by the same two independent reviewers. Any disagreements between reviewers were settled by a third reviewer.

### Data extraction

As described in the previous study^24^, two reviewers performed data extraction independently by a standardized data extraction form. If a consensus could not be reached, a third reviewer assessed the data point and made the final decision. The primary source of all data was numerical data reported in tables or figures. If the data was reported in graphical form, a graph digitizing software (Engauge digitizer 10.8, Mark Mitchel, 2014) was used to extract data. The corresponding authors of studies were tried to be contacted for insufficient data.

The following data were extracted: the author, year of publication, study location, types of anesthesia, number of patients, average ages of participants, average BMI of participants, intervention and comparator group, timing of nerve block. We also extracted measures of variance at all reported times for postoperative pain scores, postoperative analgesic consumption, time to first analgesic request and postoperative nausea and vomiting (PONV).

### Quality assessment

The Cochrane risk of bias tool which is recommended by the Cochrane Collaboration for risk of bias assessment, was used in this study^25^. There are seven domains in the Cochrane risk of bias tool, including the random sequence generation, allocation concealment, blinding of participants and personnel, blinding of outcome assessment, incomplete outcome data, selective reporting and other bias. The judgment of each domain is presented as “low risk”, “high risk” or “unclear risk” based on the instruction of Cochrane Collaboration. To assess the quality of evidence, we used the Grading of Recommendations Assessment, Development and Evaluation (GRADE) method exploring the five different GRADE domains including study limitations, consistency of effect, imprecision, indirectness and publication bias. The above assessments were performed by two reviewers with disagreement settled by a third reviewer as described in the previous study^24^.

### Primary and secondary outcomes

The primary outcomes were cumulative postoperative intravenous opioid consumption (in milligram morphine equivalents) at 24 h and 48 h. The secondary outcomes included VAS scores at rest and dynamic 2, 4, 6, 12, 24, 48 h postoperatively, the time to first request for rescue analgesic, incidence of PONV. In order to standardize analysis, all postoperative opioid analgesics were converted to equivalent morphine doses by using recognized conversion ratios^26^ and all postoperative pain scores were converted to an equivalent score on the 0–10 cm Visual Analog Scale (VAS). Any reported postoperative nausea or vomiting in the included studies was treated as PONV.

### Statistical methods

Data analysis was performed by the Review Manager software (RevMan, version 5.3.5; Nordic Cochrane Centre, The Cochrane Collaboration, Copenhagen, Denmark) and “meta” package in R Studio (Version1.1.442—©2009–2018 RStudio, Inc.). Risk ratios (RR) with corresponding 95% confidence interval (95% CI) was calculated for dichotomous data and continuous data were analysed using mean difference (MD) with corresponding 95% CI. Heterogeneity among studies was evaluated using the *I*^2^ statistic. If the *I*^2^ statistic was greater 50%, random-effect model was used, otherwise a fixed-effect model was used^27^. The above methods were in consistent with the description in our previous study^24^. Subgroup analysis was performed to evaluate pain score at different time points postoperatively and assess the effect of QLBs conducted in different approaches. Moreover, sensitivity analysis was adopted to evaluate the effect of excluding studies that were published in Chinese and in which morphine was used in spinal anesthesia. Funnel plot with Egger’s test was used to detect potential publication bias. For all tests, statistical significance was defined as a *P* < 0.05.

### Trial sequential analysis (TSA)

TSA is a statistical method that can determine whether the evidence in the meta-analysis is reliable and conclusive. We performed TSA for our primary outcomes. The required sample size was calculated to determine whether the evidence in our meta-analysis is reliable and conclusive based on the observed data and the trial sequential monitoring boundaries (TSMB). If the overall sample size in the meta-analysis reaches the required sample size, or the cumulative Z-value curve passes through the sequential monitoring boundary of the test or enters the invalid region, the results of meta-analysis are likely to be stable and no further testing is needed. Otherwise, it indicates insufficient evidence to reach a conclusion and further research is needed^28^.

The diversity-adjusted information size and O’Brien–Flemingα-spending boundaries were calculated using 2-sided 5% type I error (alpha of 5%) and 20% type 2 error (beta of 20%) rate (80% power), and the mean difference and variance were calculated from the low risk of bias studies. The heterogeneity correction was based on model variance. The software TSA version 0.9.5.10 beta was used for these analyses.

## Results

**Figure 1:**
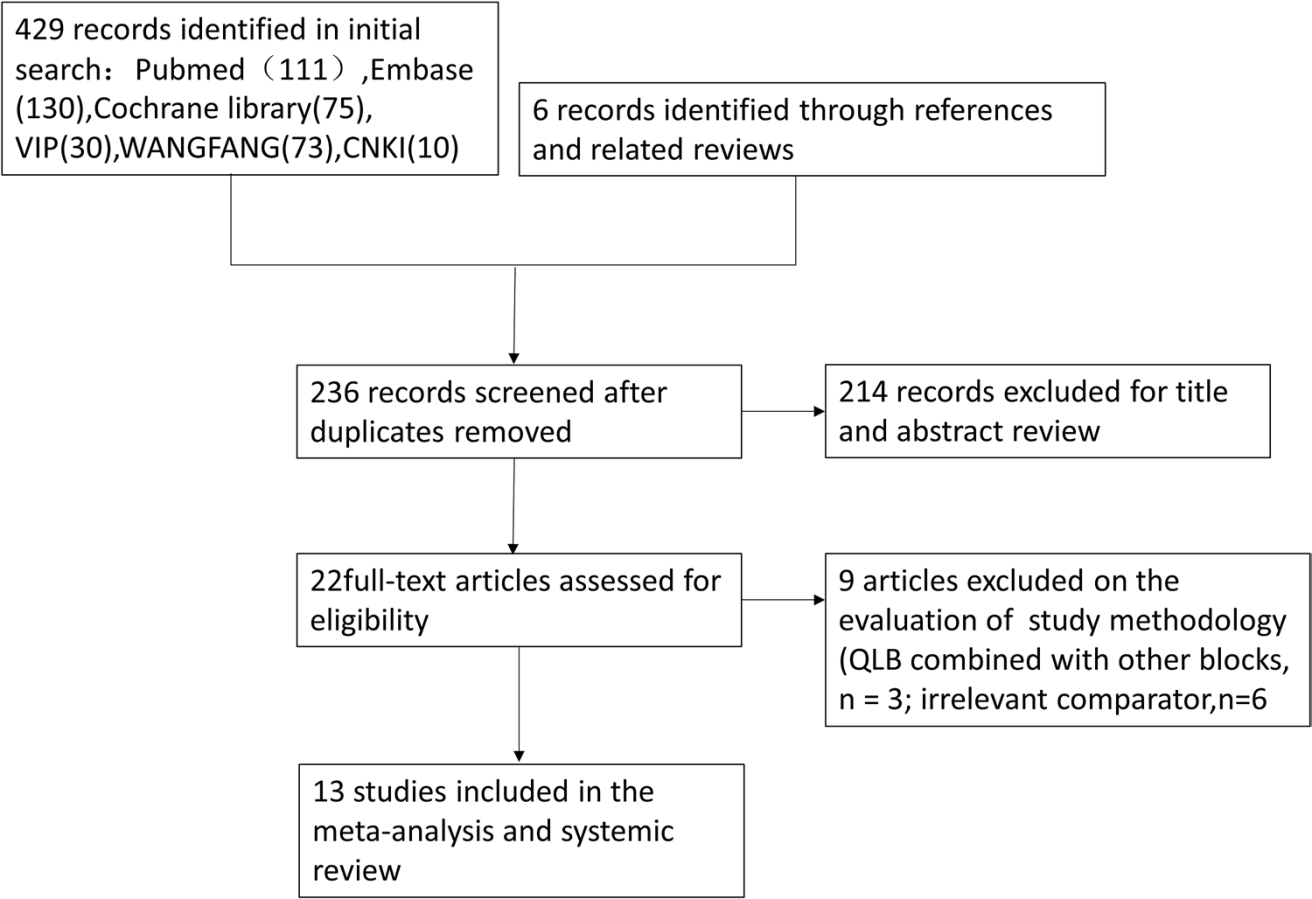
The flow diagram of the study.

A total of 236 unique citations were identified by our initial search strategy after duplicate articles were removed. The full-text versions of 22 potentially eligible citations were retrieved after exclusion of 214 impertinent studies based on title and abstract screening. Of these studies, 9 were excluded for the following reasons: incorrect intervention (QLB combined with other blocks, n = 3), and irrelevant comparator (TAP or local anesthetic infiltration, n = 6). Finally, a total of 13 randomized controlled trials^9–20,29^ were included in this meta-analysis. The flow diagram of study selection is shown in Fig. 1.

### Description of included studies

The characteristics of included studies in this review are presented in Table 1. The 13 randomized controlled trials involved a total of 1269 patients, of which 632 received QLB, and 637 received systemic analgesia alone (i.e., no block or sham block, as Control). The CS was conducted under spinal anesthesia with bupivacaine in 10 studies^9–12,15,18–20^. Of these studies, fentanyl 10–20 μg, morphine 0.1 mg and sufentanil 2.5–4 μg was respectively added to the bupivacaine solution in 5 studies^9,11,12,19,20^, 2 studies^19,20^ and 2 studies^10,29^. The combined spinal and epidural analgesia was performed in two studies and general anesthesia was adopted in only one studies for CS. The posterior QLB was conducted in 7 studies, the lateral QLB was performed in 3 studies and the transmuscular QLB was adopted in 2 studies. The QLB was performed following the completion of the CS in all studies. Sham block with equal volume of 0.9% saline was performed in 6 studies and no block was performed in the other studies as control. An included study by Tamura et al. had four groups (two active and two controls) and the individual values for each group were reported separately. Therefore, we labelled these two comparison arms as ^(a)^ and ^(b)^ for a better reader comprehension.

**Table 1:** Characteristics of included studies.

Study ID	Country	Anesthesia	Approach	Number per group		Age		Intervention time point	BMI		Intervention drugs	
				QLB	CON	QLB	CON		QLB	CON	QLB	CON
Blanco^9^	United Arab Emirates	Spinal anaesthesia with hyperbaric bupivacaine 15 mg and fentanyl 20 mg	Posterior QLB	25	23	47.6 ± 12.8	46.4 ± 13.8	At the end of surgery	NR	NR	0.125% bupivacaine 0.2 ml/kg on each side	0.9% normal saline 0.2 ml/kg on each side
Hansen^29^	Denmark	Spinal anesthesia with hyperbaric bupivacaine 10 mg and sufentanil 2.5 µg	Transmuscular QLB	34	34	32.3 ± 5.7	31.5 ± 4.9	Following completion of the surgery	31.2 ± 5.5	30.2 ± 3.4	30 ml of ropivacaine 0.375% on each side	30 ml of saline 0.9% on each side
He^19^	China	Epidural anesthesia with 2% lidocaine 60–100 mg	Lateral QLB	30	30	28.3 ± 2.9	27.1 ± 3.2	Following completion of the surgery	NR	NR	0.33% ropivacaine 20 ml on each side	None
Krohg^10^	Switzerland	Spinal anesthesia with isobaric bupivacaine 10 mg and sufentanil 4 μg	Lateral QLB	20	20	34 ± 4	36 ± 4	Within the first hour after cesarean delivery	26 ± 3	28 ± 3	0.2% ropivacaine 0.4 ml/kg with a maximum of 30 ml on each side	0.9% saline 0.4 ml/kg with a maximum of 30 ml on each side
Shan^15^	China	Spinal anesthesia with 0.5% bupivacaine 12 mg	Transmuscular QLB	30	30	27 ± 4	28 ± 3	Following completion of the surgery	NR	NR	0.25% ropivacaine 0.5 ml/kg on each side	None
Tamura^20^ ^a^	Japan	Spinal anesthesia with 0.5% hyperbaric bupivacaine 11–13 mg and fentanyl 10 μg and morphine 0.1 mg	Posterior QLB	34	38	35.2 ± 4.2	33.7 ± 5.8	Immediately after surgery	NR	NR	0.3% ropivacaine 0.45 ml/kg each sideup to a maximum of 75 mg	Saline 0.45 ml/kg each side
Tamura^20^ ^b^	Japan	Spinal anesthesia with hyperbaric bupivacaine 0.5% 11–13 mg and fentanyl 10 μg	Posterior QLB	36	38	33.2 ± 4.8	35.3 ± 4.8	Immediately after surgery	NR	NR	0.3% ropivacaine 0.45 ml/kg each sideup to a maximum of 75 mg	Saline 0.45 ml/kg each side
Zhang^17^	China	Combined spinal and epidural analgesia	Posterior QLB	30	30	32.1 ± 4.1	32.5 ± 4.8	After surgery	NR	NR	0.25% ropivacaine 30 ml each side	None
Zhang^16^	China	General anesthesia	Posterior QLB	25	25	29.2 ± 0.8	28.5 ± 0.5	After surgery	38.4 ± 0.2	38.9 ± 0.2	0.3% ropivacaine 25 ml on each side	None
Irwin^19^	Ireland	Spinal anesthesia using hyperbaric bupivacaine 0.5% 2.0–2.3 ml including morphine 0.1 mg and fentanyl 20 μg	Posterior QLB	44	42	35 ± 4	33 ± 5	After surgery	27 ± 4	26 ± 4	0.25% revobupivacaine 20 ml injected on each side	None
Salama^12^	Egypt	Spinal anesthesia with 12.5 mg of hyperbaric bupivacaine 0.5% and fentanyl 10 µg	Posterior QLB	30	30	31.09 ± 5.87	32.49 ± 6.57	After surgery	29.17 ± 6.17	29.63 ± 6.74	24 ml of 0.375% ropivacaine on each side	Same volume of 0.9% saline
Mieszkowski^11^	Poland	Spinal anesthesia i with 12.5 mg of hyperbaric bupivacaine 0.5% and fentanyl 20 µg	Lateral QLB	28	30	28.75 ± 3.25	29.29 ± 4.55	After wound closure	30.43 ± 4.09	30.63 ± 4.85	24 ml of 0.375% ropivacaine per side (in total 180 mg)	None
Wang^18^	China	Spinal anesthesia with 10 mg of hyperbaric bupivacaine 0.5%	Lateral QLB	35	35	26.4 ± 4.1	26.9 ± 3.8	After wound closure	29.1 ± 1.7	29.5 ± 1.9	24 ml of 0.375% ropivacaine per side (in total 180 mg)	Same volume of 0.9% saline
Cai^13^	China	Combined spinal and epidural analgesia	Posterior QLB	231	232	29.52 ± 7.48	29.99 ± 7.45	After surgery	NR	NR	30 ml of 0.25% ropivacaine per side	None

*QLB* quadratus lumborum block, *CON* control, *BMI* body mass index, *NR* not reported.

### Risk of bias assessment

**Figure 2:**
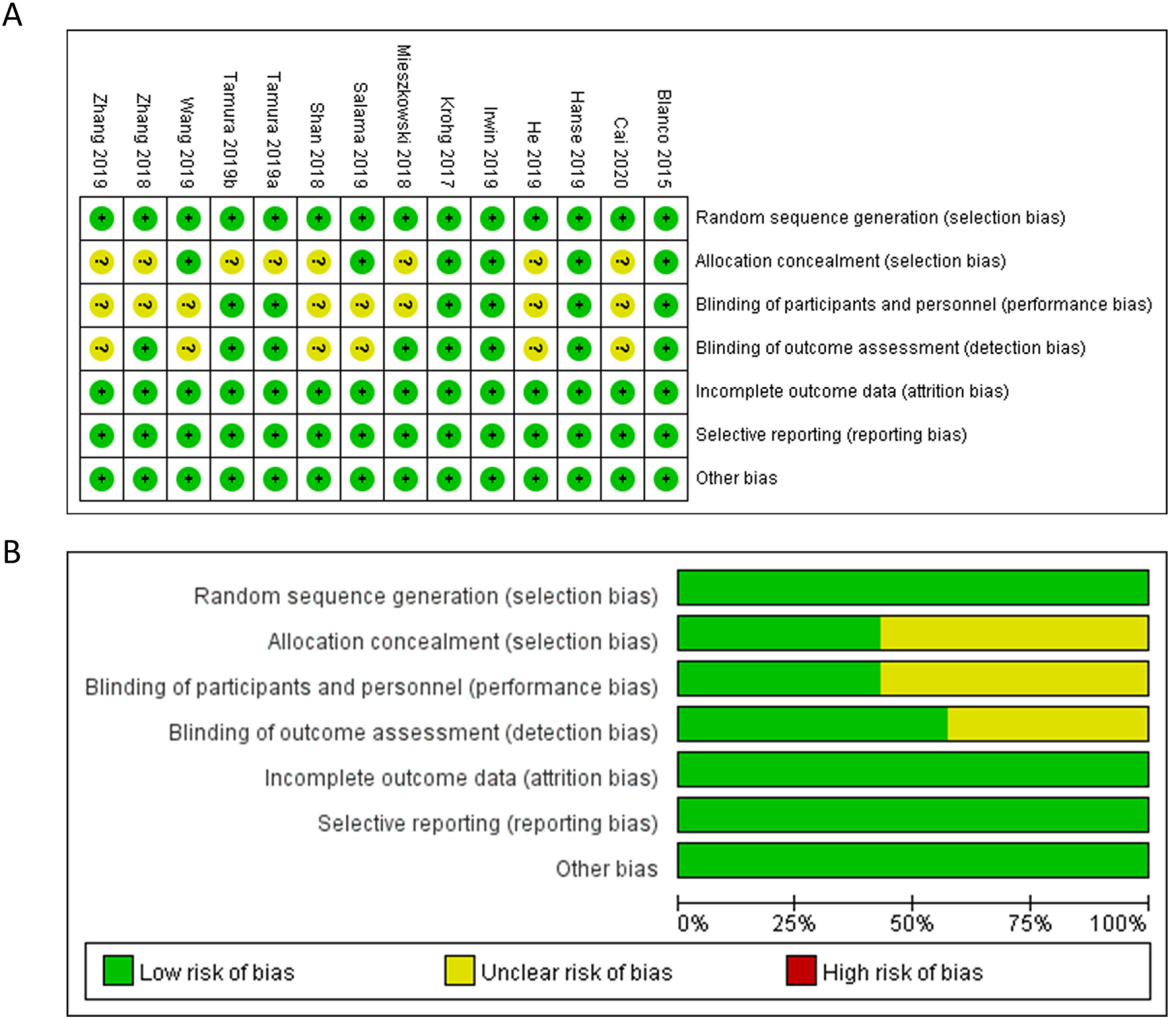
Quality assessment of included studies. The green circles indicate lack of bias; yellow circles indicate unclear bias. (**A**) Risk of bias for each included study. (**B**) The overall summary of bias of the included studies.

The risk of bias assessment of the included studies is presented in Fig. 2. All of the included studies provided satisfactory description of random sequence generation (low risk of selection bias). Eight studies did not provide sufficient information about allocation concealment (unclear risk of selection bias). Moreover, eight studies did not explicitly state the blinding process of participants and personnel (unclear risk of performance bias). Six studies did not explicitly state the blinding process of outcome assessment (unclear risk of detection bias). All of the included studies reported the complete outcome data (low risk of attrition bias) and unclear risk of other bias were found in all studies included.

### Cumulative 24-h intravenous morphine equivalent consumption

**Figure 3:**
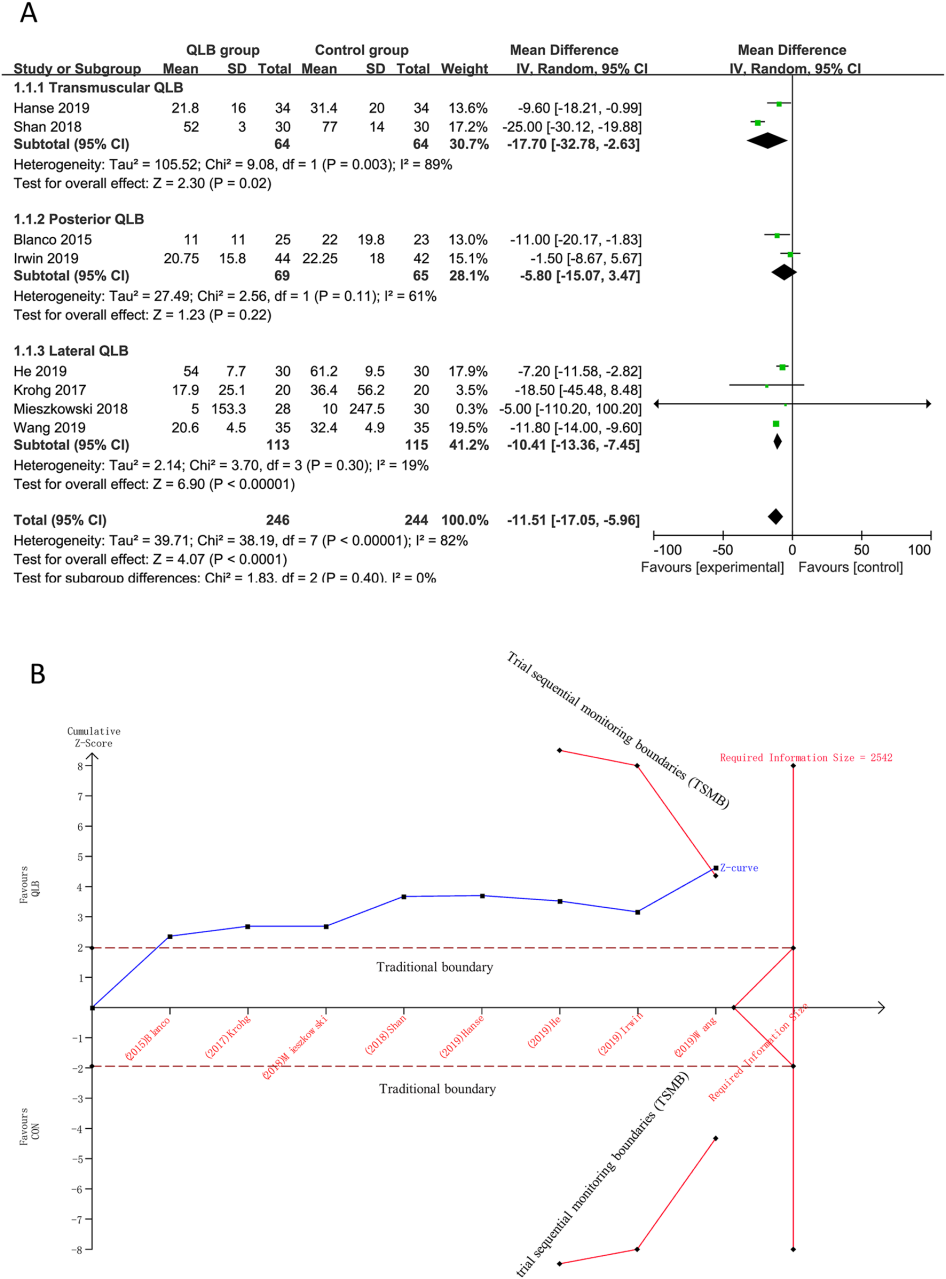
The results of meta-analysis and TSA for 24 h intravenous morphine equivalent consumption. (**A**) meta-analysis of cumulative 24-h morphine equivalent consumption; (**B**) TSA of cumulative 24-h morphine equivalent consumption.

Eight studies (490 participants; QLB: 246, Control: 244) that reported cumulative 24-h intravenous morphine equivalent consumption provided sufficient data for statistical pooling. Overall, QLB significantly reduced the cumulative 24-h intravenous morphine equivalent consumption compared with Control (MD, − 11.51 mg; 95% CI − 17.05 to − 5.96, *I*^2^ = 82%; *P* < 0.01) (Fig. 3A).

Subgroup analysis in the settings of transmuscular QLB and lateral QLB showed statistically significant effect of QLB in reducing the cumulative 24-h intravenous morphine equivalent consumption compared with Control (Transmuscular QLB VS. Control: MD, − 17.70 mg; 95% CI − 32.78 to − 2.63, *I*^2^ = 89%, *P* < 0.05; Lateral QLB VS. Control: MD, − 10.41 mg; 95% CI − 13.36 to − 7.45, *I*^2^ = 19%, *P* < 0.01). However, sub-group analysis in the settings of posterior QLB showed no statistically significant effect of QLB in reducing the cumulative 24-h intravenous morphine equivalent consumption (MD, − 5.80 mg; 95% CI − 15.07 to 3.47, *I*^2^ = 61%; P = 0.22) (Fig. 3A).

300 participants (QLB: 151, Control: 149) and 404 participants (QLB: 202, Control: 202) were respectively included in sensitivity analysis of excluding studies that were published in Chinese and studies with morphine in spinal anesthesia. The sensitivity analyses supported the conclusion that QLB significantly reduced the increase cumulative 24-h intravenous morphine equivalent consumption (MD, − 6.81 mg; 95% CI − 11.46 to − 2.17, *I*^2^ = 1%; *P* < 0.01; MD, − 13.28 mg, 95% CI − 18.97 to − 7.58, *I*^2^ = 80%; *P* < 0.01, respectively) (Table S1).

The minimal clinical significance value estimated from the low risk of bias studies was 7.7 mg and the TSA results showed that a diversity-adjusted required information size (RIS) of 2542 patients was calculated. Although the RIS was not reached, the cumulative Z-value curve crossed both the traditional boundary and the trial sequential monitoring boundaries (TSMB) which indicated that the result of the meta-analysis is stable (Fig. 3B).

### Cumulative 48-h intravenous morphine equivalent consumption

**Figure 4:**
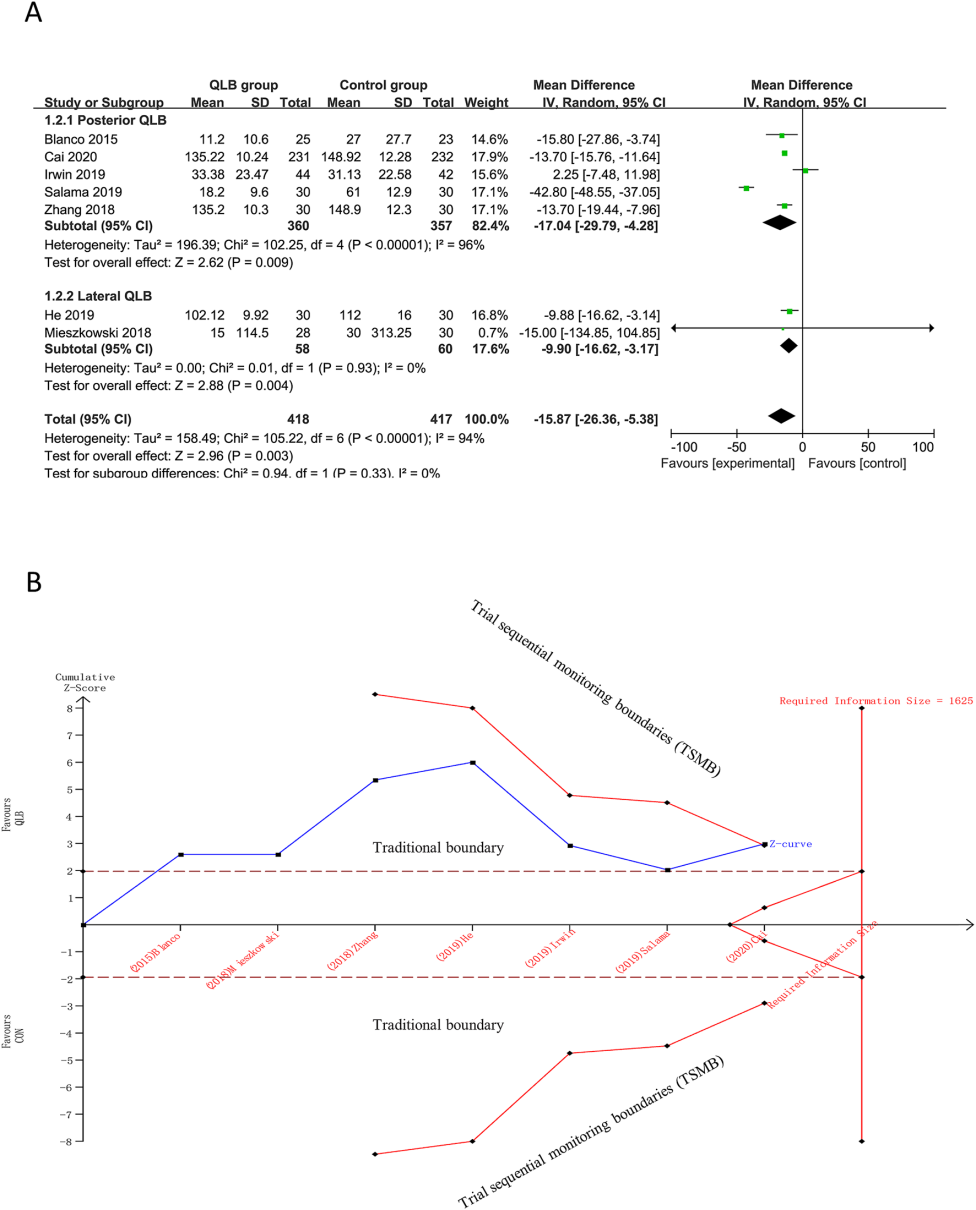
The results of meta-analysis and TSA for 48 h intravenous morphine equivalent consumption. (**A**) meta-analysis of cumulative 48-h morphine equivalent consumption; (**B**) TSA of cumulative 48-h morphine equivalent consumption.

Seven studies (835 participants; QLB: 418, Control: 417) that reported cumulative 48-h intravenous morphine equivalent consumption were available for statistical pooling. Overall, QLB significantly reduced the cumulative 48-h intravenous morphine equivalent consumption compared with Control (MD, − 15.87 mg; 95% CI − 26.36 to − 5.38, *I*^2^ = 94%; *P* < 0.01) (Fig. 4A).

Subgroup analysis in the settings of both posterior QLB and lateral QLB showed statistically significant effect of QLB in reducing the cumulative 48-h intravenous morphine equivalent consumption compared with Control [Posterior QLB VS. Control: MD, − 17.04 mg; 95% CI − 29.79 to − 4.28, *I*^2^ = 96%, *P* < 0.01; Lateral QLB VS. Control: MD, − 9.90 mg; 95% CI − 16.62 to − 3.17, *I*^2^ = 0%, *P* < 0.01] (Fig. 4A).

749 participants (QLB: 374, Control: 375) were included in sensitivity analysis of excluding studies without morphine in spinal anesthesia and the result of the sensitivity analysis showed significant reduction of cumulative 48-h intravenous morphine equivalent consumption (MD, − 19.23 mg; 95% CI − 30.49 to − 7.97, *I*^2^ = 95%, *P* < 0.01). However, the sensitivity analysis of excluding studies that were published in Chinese did not suggested QLB significantly reduced the cumulative 48-h intravenous morphine equivalent consumption (QLB: 127, Control: 125, MD, − 18.8 mg; 95% CI − 47.5 to 9.9, *I*^2^ = 95%; *P* = 0.2) (Table S1).

The minimal clinical significance MD estimated from the low risk of bias studies was 20 mg and the TSA results showed that a diversity-adjusted RIS of 1625 patients was calculated. Although the RIS was not reached, the cumulative Z-value curve crossed both the traditional boundary and the TSMB, suggesting that the result of the meta-analysis is stable (Fig. 4B).

### Postoperative pain score at rest

**Figure 5:**
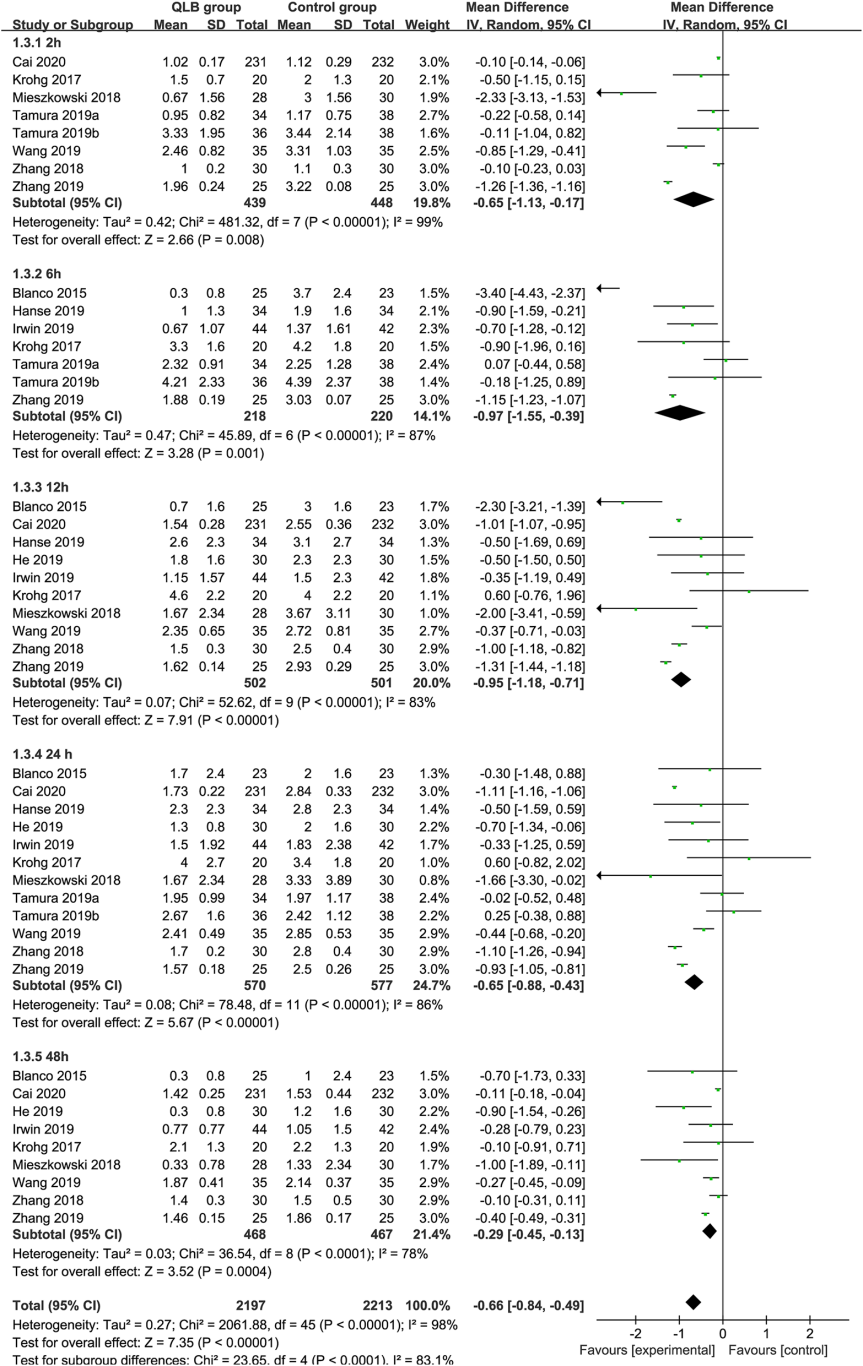
Forest plots of pain scores at rest at different time points after surgery at rest.

Comparing QLB with Control for postoperative pain scores at rest, the number of participants included at each time point was 887 (QLB:439, Control: 448), 438(QLB:218, Control: 220), 1003 (QLB:502, Control: 501), 1147 (QLB:570, Control: 577), 539 (QLB:468, Control: 467) at 2, 6, 12, 24 and 48 h, respectively. Compared with Control, QLB improved pain control at 2, 6, 12, 24 and 48 h postoperatively, by a mean difference [99% CI] equivalent to − 0.65 [− 1.13, − 0.17] (*P* < 0.01, *I*^2^ = 99%), − 0.97 [− 1.55, − 0.39] (*P* < 0.01, *I*^2^ = 87%), − 0.95 [− 1.18, − 0.71] (*P* < 0.01, *I*^2^ = 83%), − 0.65 [− 0.88, − 0.43] (*P* < 0.01, *I*^2^ = 86%), − 0.29 [− 0.45, − 0.13] (*P* < 0.01, *I*^2^ = 78%), respectively. The overall effect of meta-analysis showed that QLB significantly reduced postoperative pain scores at rest between compared with Control (MD = − 0.66, 95% CI − 0.84 to − 0.49,* P* < 0.01, *I*^2^ = 98%) (Fig. 5).

### Postoperative pain score during movement

**Figure 6:**
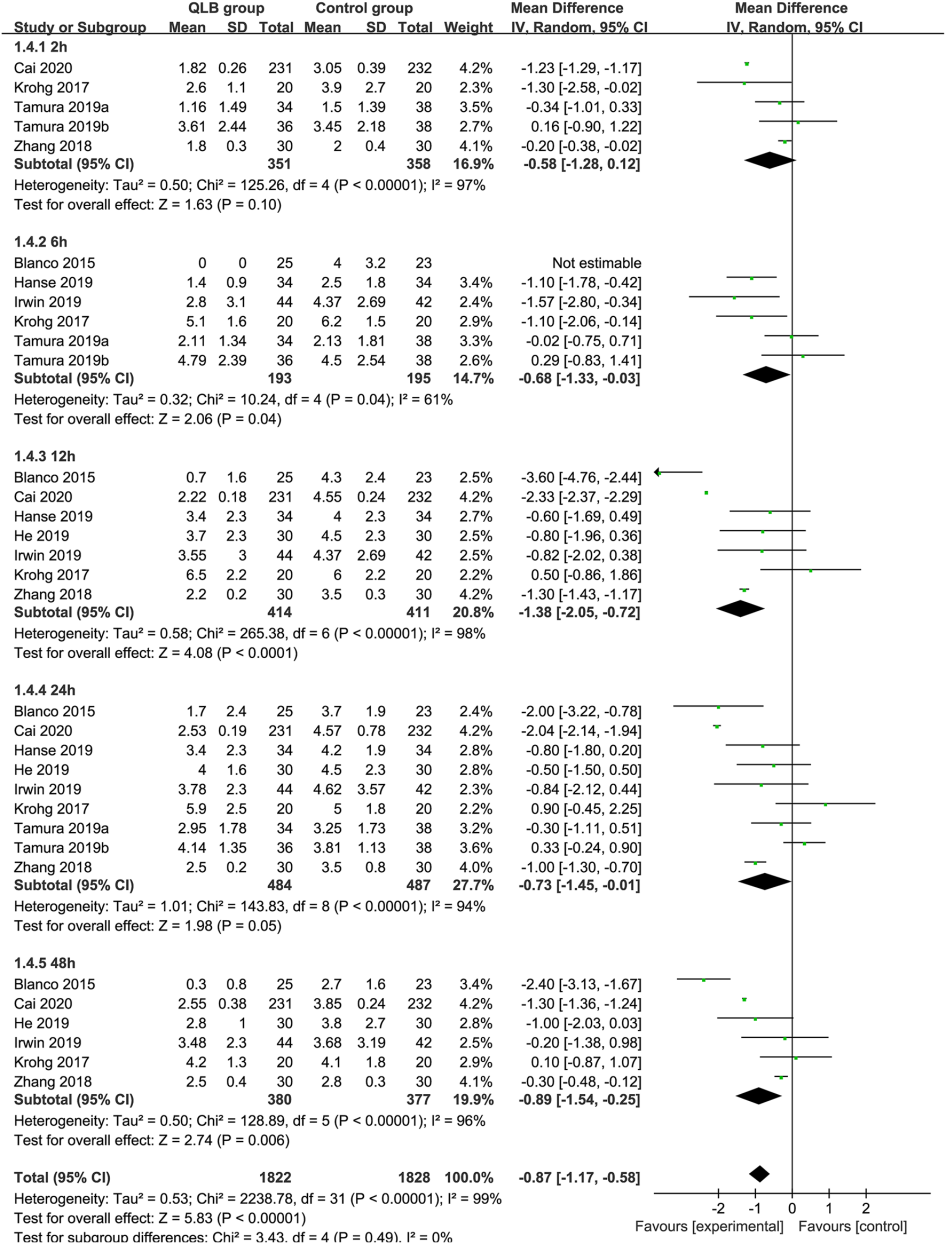
Forest plots of pain scores during movement at different time points after surgery.

Comparing QLB with Control for postoperative pain scores at movement, the number of participants included at each time point was 709 (QLB:351, Control: 358), 388 (QLB:193, Control: 195), 825 (QLB:414, Control: 411), 971 (QLB:484, Control: 487), 757 (QLB:380, Control: 377) at 2, 6, 12, 24 and 48 h, respectively. Compared with Control, QLB improved pain control at 6, 12, 24 and 48 h postoperatively, by a mean difference [99% CI] equivalent to − 0.68 [− 1.33, − 0.03] (*P* < 0.05, *I*^2^ = 61%), − 1.38 [− 2.05, − 0.72] (*P* < 0.01, *I*^2^ = 98%),− 0.73 [− 1.45, − 0.01] (*P* < 0.01, *I*^2^ = 94%), − 0.89 [− 1.54, − 0.25] (*P* < 0.01, *I*^2^ = 96%), respectively. However, QLB did not showed significant effect in reducing pain score during movement at 2 h postoperatively (MD = −0.58, 95% CI − 1.28 to 0.12, *P* = 0.1, *I*^2^ = 97%). The overall effect of meta-analysis showed that QLB significantly reduced postoperative pain scores during movement compared with control (MD = − 0.87, 95% CI − 1.17 to − 0.58,* P* < 0.01, *I*^2^ = 99%) (Fig. 6).

### Time to first analgesic request

For QLB versus Control, the time to first analgesic request was reported in five studies (709 patients; QLB: 353, Control: 356). Compared with Control, patients receiving QLB had a longer time to first analgesic request, by 8.37 h [0.19, 16.54] (*P* < 0.05, *I*^2^ = 100%) (Fig. S1).

### Incidence of postoperative PONV

Nine studies (965 participants; QLB:484, Control:481) reported the incidence of postoperative PONV. The result of meta-analysis showed that QLB significantly reduced the incidence of PONV compared with Control (RR = 0.56, 95% CI 0.37 to 0.83, *P* < 0.01, *I*^2^ = 48%) (Fig. S2).

### Publication bias

We assessed publication bias by funnel plots with Egger’s test. The funnel plot of each outcome was presented in Fig. 3S and Fig. 4S. The publication bias was further quantified using the Egger’s test. Significant publication bias was indicated in postoperative pain score at rest (*P* < 0.01) and during movement (*P* = 0.02) 24 h postoperatively. Significant publication bias is unlikely for the other outcomes with *P* > 0.05.

### Quality of evidence

For each included outcome, quality of the evidence synthesized using the GRADE approach was shown in Table 2. Overall, most quality of the evidence of the included outcomes were moderate and low.

**Table 2:** Quality assessment of reported results by GRADE method.

Quality assessment							No of patients		Effect		Quality	Importance
No of studies	Design	Risk of bias	Inconsistency	Indirectness	Imprecision	Other considerations	QLB	CON	Relative (95% CI)	Absolute		
**Cumulative 24-h intravenous morphine equivalent consumption (better indicated by lower values)**												
8	Randomised trials	No serious risk of bias^a^	Serious	No serious indirectness	No serious imprecision	None	246	244	–	MD 11.51 lower (17.05–5.96 lower)	⊕⊕⊕O Moderate	
**Cumulative 24-h intravenous morphine equivalent consumption—transmuscular QLB (better indicated by lower values)**												
2	Randomised trials	No serious risk of bias	Serious	No serious indirectness	Serious	None	64	64	–	MD 17.7 lower (32.78–2.63 lower)	⊕⊕OO Low	
**Cumulative 24-h intravenous morphine equivalent consumption—posterior QLB (better indicated by lower values)**												
2	Randomised trials	No serious risk of bias	Serious	No serious indirectness	Serious	None	69	65	–	MD 5.8 lower (15.07 lower–3.47 higher)	⊕⊕OO Low	
**Cumulative 24-h intravenous morphine equivalent consumption—lateral QLB (better indicated by lower values)**												
4	Randomised trials	No serious risk of bias	No serious inconsistency	No serious indirectness	Serious	None	113	115	–	MD 10.41 lower (13.36–7.45 lower)	⊕⊕⊕O Moderate	
**Cumulative 48-h intravenous morphine equivalent consumption (better indicated by lower values)**												
7	Randomised trials	No serious risk of bias	Serious^a^	No serious indirectness	No serious imprecision	None	418	417	–	MD 15.87 lower (26.36–5.38 lower)	⊕⊕⊕O Moderate	
**Cumulative 48-h intravenous morphine equivalent consumption—posterior QLB (better indicated by lower values)**												
5	Randomised trials	No serious risk of bias	Serious	No serious indirectness	No serious imprecision	None	360	357	–	MD 17.04 lower (29.79–4.28 lower)	⊕⊕⊕O Moderate	
**Cumulative 48-h intravenous morphine equivalent consumption—lateral QLB (better indicated by lower values)**												
2	Randomised trials	No serious risk of bias	No serious inconsistency	No serious indirectness	Serious	None	58	60	–	MD 9.9 lower (16.62–3.17 lower)	⊕⊕⊕O Moderate	
**VAS at res (better indicated by lower values)**												
12	Randomised trials	No serious risk of bias	Serious	No serious indirectness	No serious imprecision	None	2197	2213	–	MD 0.66 lower (0.84–0.49 lower)	⊕⊕⊕O Moderate	
**VAS at res—2 h (better indicated by lower values)**												
8	Randomised trials	No serious risk of bias	Serious	No serious indirectness	No serious imprecision	None	439	448	–	MD 0.65 lower (1.13–0.17 lower)	⊕⊕⊕O Moderate	
**VAS at res—6 h (better indicated by lower values)**												
7	Randomised trials	No serious risk of bias	Serious	No serious indirectness	No serious imprecision	None	218	220	–	MD 0.97 lower (1.55–0.39 lower)	⊕⊕⊕O Moderate	
**VAS at res—12 h (better indicated by lower values)**												
10	Randomised trials	No serious risk of bias	Serious	No serious indirectness	No serious imprecision	None	502	501	–	MD 0.95 lower (1.18–0.71 lower)	⊕⊕⊕O Moderate	
**VAS at res—24 h (better indicated by lower values)**												
12	Randomised trials	No serious risk of bias	Serious	No serious indirectness	No serious imprecision	None	570	577	–	MD 0.65 lower (0.88–0.43 lower)	⊕⊕⊕O Moderate	
**VAS at res—48 h (better indicated by lower values)**												
9	Randomised trials	No serious risk of bias	Serious	No serious indirectness	No serious imprecision	None	468	467	–	MD 0.29 lower (0.45–0.13 lower)	⊕⊕⊕O Moderate	
**VAS dynamic (better indicated by lower values)**												
9	Randomised trials	No serious risk of bias	Serious	No serious indirectness	No serious imprecision	None	1822	1828	–	MD 0.87 lower (1.17–0.58 lower)	⊕⊕⊕O Moderate	
**VAS dynamic—2 h (better indicated by lower values)**												
5	Randomised trials	No serious risk of bias	No serious inconsistency^a^	No serious indirectness	Serious	None	351	358	–	MD 0.58 lower (1.28 lower–0.12 higher)	⊕⊕⊕O Moderate	
**VAS dynamic—6 h (better indicated by lower values)**												
6	Randomised trials	No serious risk of bias	Serious	No serious indirectness	Serious	None	193	195	–	MD 0.68 lower (1.33–0.03 lower)	⊕⊕OO Low	
**VAS dynamic—12 h (better indicated by lower values)**												
7	Randomised trials	No serious risk of bias	Serious	No serious indirectness	No serious imprecision	None	414	411	–	MD 1.38 lower (2.05–0.72 lower)	⊕⊕⊕O Moderate	
**VAS dynamic—24 h (better indicated by lower values)**												
9	Randomised trials	No serious risk of bias	Serious	No serious indirectness	No serious imprecision	`None	484	487	–	MD 0.73 lower (1.45–0.01 lower)	⊕⊕⊕O Moderate	
**VAS dynamic—48 h (better indicated by lower values)**												
6	Randomised trials	No serious risk of bias	Serious	No serious indirectness	No serious imprecision	None	380	377	–	MD 0.89 lower (1.54–0.25 lower)	⊕⊕⊕O Moderate	
**Incidence of PONV**												
9	Randomised trials	No serious risk of bias	No serious inconsistency	No serious indirectness	No serious imprecision	None	33/484 (6.8%)	59/48,159/481 (12.3%)	OR 0.51 (0.32–0.8)	56 fewer per 1000 (from 22 to 80 fewer)	⊕⊕⊕⊕ High	
								15%		67 fewer per 1000 (from 26 to 97 fewer)		
**Time to first opioid in hours (better indicated by lower values)**												
5	Randomised trials	No serious risk of bias	Very serious	No serious indirectness	No serious imprecision	None	353	356	–	MD 8.37 higher (0.19–16.54 higher)	⊕⊕OO Low	

## Discussion

This is a meta-analysis with TSA of 13 RCTs to evaluate clinical role of QLB after CS. Our meta-analysis showed that QLB can reduce the cumulative 24-h and 48-h intravenous morphine equivalent consumption after CS. Meanwhile, the TSA further strengthened the above results and indicated no further study is needed. Moreover, our study showed that QLB significantly improved pain control at 2, 6, 12, 24 and 48 h postoperatively, extended the time to first analgesic request and reduced the incidence of PONV when compared with Control.

The analgesic efficacy of QLB is closely related to the injection position of the needle tip. There are mainly three different types of QLB according to the injection position of the needle tip, namely lateral (type 1 QLB, needle is located at the lateral margin of the quadratus lumborum muscle), posterior (type 2 QLB, needle is located at the posterior border of the quadratus lumborum muscle), and transmuscular approaches (type 3 QLB, needle is located the anterior border of the quadratus lumborum muscle). It was reported that local anesthetic spread mainly to the transversus abdominis muscle plane in QLB1, along the middle thoracolumbar fascia in QLB2, and into the thoracic paravertebral space to in QLB3^30^. Our subgroup analysis showed that transmuscular QLB and lateral QLB significantly reduced the cumulative 24-h intravenous morphine equivalent consumption compared with Control. However, posterior QLB showed no statistically significant effect of QLB in reducing the cumulative 24-h intravenous morphine equivalent consumption. There are two potential reasons to explain the above results. One is that the uncertain anatomical structure of connective tissue and relative resistance to the spread of local anesthetic makes it difficult to insure the spread of QLB2 block anesthetic even in the guidance of ultrasound^31^, which may lead to the insignificant effect in reducing the cumulative 24-h intravenous morphine equivalent consumption. The other one is that there are only two studies included in the subgroup analysis of QLB2 and the QLB2 was performed in conjunction with intrathecal morphine in one of the included studies^19^, which may also lead to the insignificant effect. It has been reported that TAP did not showed additional analgesic effect in CS patients when intrathecal morphine is administrated. Similar results have been reported in QLB. There are two trials^19,20^ combined intrathecal morphine with QLB in the current study and both of them showed insignificant analgesic effect compared with Control. Our sensitivity analysis of excluding studies with morphine in spinal anesthesia did not changed the overall effect of QLB. Moreover, the TSA further clarified the conclusions of our primary outcome that QLB significantly reduces the cumulative 24-h and 48-h intravenous morphine equivalent consumption after CS.

The significant reduction in postoperative pain score and time to first analgesic request could be mainly explained by the reliable analgesic effect of QLB through sensory blockade from T7 to L1. However, QLB did not showed significant effect in reducing pain score during movement at 2 h postoperatively, which may be attributed to the residual analgesic effects of spinal anesthesia. In addition, the current study showed reduced incidence of PONV in QLB which may be associated with the decrease in the use of opioid analgesics after surgery.

There are several strengths in the current study. Firstly, we conducted a systematic on the common used Chinese and international databases. It is necessary to include studies published in Chinese in order to make a systematic assessment of the role of QLB in CS as China is the world's most populous country. Secondly, the systematic methodology was used in our current study to identify the trials and evaluation of their quality of evidence. We adopted the Cochrane risk of bias tool and the GRADE method to identify the risks of included trials and assess the quality of evidence of our findings. Thirdly, we performed a subgroup analysis to identify the analgesic effects of different approaches of QLB, which was never achieved in the previous studies. Furthermore, we were capable of eliminating the possibility of false-positive result for our primary outcome (morphine consumption at 24 h and 48 h) by using TSA.

Several limitations should also be noted in our study. First of all, heterogeneity of the included studies must be considered. Trials included in most analyses of the current study showed significant heterogeneity mainly due to differences in volumes and dosage of local anaesthetic drugs and variations in application of postoperative multimodal analgesia. Moreover, the effect of systemic analgesia administered during general anasthesia may also contributed to the heterogeneity. However, there is only one trial conducted under general anesthesia in our study, and therefore the effect on our results may be slight. Secondly, evaluation of the success ratio of QLB was not performed in most of the included studies, thus may affected the results of our analysis.

In conclusion, the results of this meta-analysis with TSA of QLB compared with inactive control for analgesia following caesarean delivery suggest that QLB provide better opioid-sparing effect at 24-h and 48-h postoperatively. The TSA ruled out the possibility of false-positive thus further strengthened the above results. Moreover, our study showed reduced postoperative pain score, time to first analgesic request and incidence of PONV in QLB compared with inactive control. However, the evidence quality of most results are low and modest, therefore, these conclusions should be interpreted with caution.

## Supplementary Information

Supplementary Information.
