# Supplementary material for: Quadratus lumborum block for postoperative analgesia after cesarean section: a meta-analysis of randomized controlled trials with trial sequential analysis
Source: Sci Rep. 2021 Sep 13;11:18104. doi: 10.1038/s41598-021-96546-7 (PMC8438068; doi:10.1038/s41598-021-96546-7)
Supplement: Supplementary file 1 — Supplementary Information. [file 41598_2021_96546_MOESM1_ESM.docx]

**Quadratus lumborum block for postoperative** **analgesia after cesarean section: a meta-analysis of randomized controlled trials with trial sequential analysis.**

Zhigang Zhao^1,2^, MD, Kaiming Xu^1^, MD, Yanting Zhang^3^, MD, Gang Chen^3^, MD, Youfa Zhou^3*^, MD.

1. Department of Anesthesiology, Shaoxing Campus, Sir Run Run Shaw Hospital, School of Medicine, Zhejiang University, Shaoxing, China
2. Department of Anesthesiology, Shaoxing Shangyu Second Peple’s Hospital, Shaoxing, China
3. Department of Anesthesiology, Sir Run Run Shaw Hospital, School of Medicine, Zhejiang University, Hangzhou, China

***Correspondence**:

Youfa Zhou, MD, Department of Anesthesiology, Sir Run Run Shaw Hospital, School of Medicine, Zhejiang University, Qingchun East Road No. 3, Hangzhou 310020, China (E-mail: youfa_zhou@zju.edu.cn, Fax: 0571-86044817, phone number: 8613615719892)

**Supplementary Table 1.** Sensitivity analysis of primary outcome.

| Outcome | Sensitivity analyses | Studies (n) | QLB | Control | RR or MD | 95% CI | P value for effect | *I^2^* |
| --- | --- | --- | --- | --- | --- | --- | --- | --- |
| Cumulative 24-h intravenous morphine equivalent consumption | Excluding studies published in Chinese | 5 | 151 | 149 | -6.81 | -11.46 to- 2.17 | <0.01 | 1% |
|  | Excluding studies with morphine in spinal anesthesia | 7 | 202 | 202 | -13.28 | -18.97 to -7.58 | <0.01 | 80% |
| Cumulative 48-h intravenous morphine equivalent consumption | Excluding studies published in Chinese | 4 | 127 | 125 | -18.8 | -47.5 to 9.9 | 0.2 | 95% |
|  | Excluding studies with morphine in spinal anesthesia | 6 | 374 | 375 | -19.23 | -30.49 to -7.97 | <0.01 | 95% |

QLB = quadratus lumborum block; (n) = the number of cases; RR = risk ratio; MD = weighted mean difference; CI = confidence interval


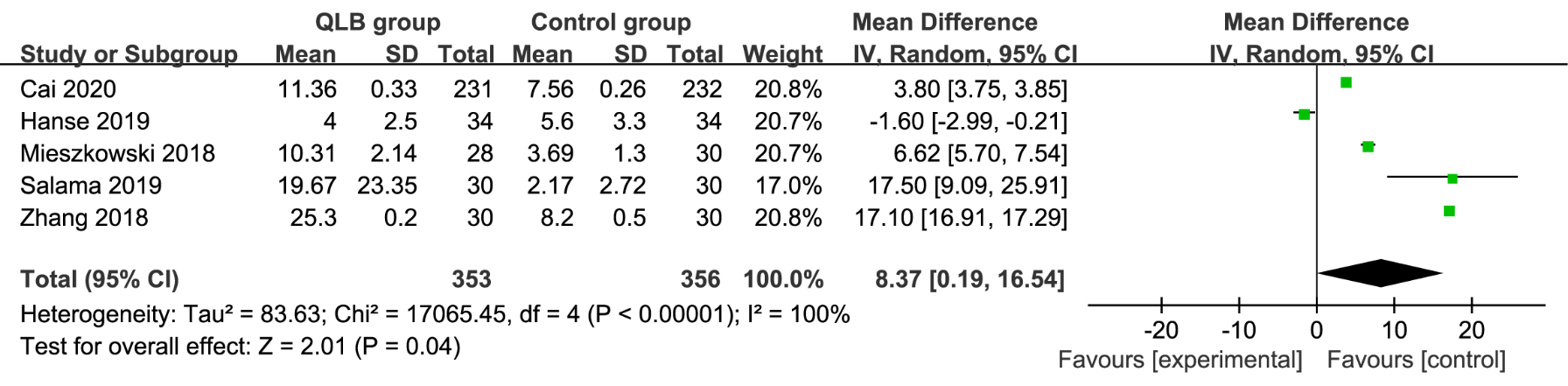
**Supplementary Figure 1.** Forest plot of time to first analgesic request


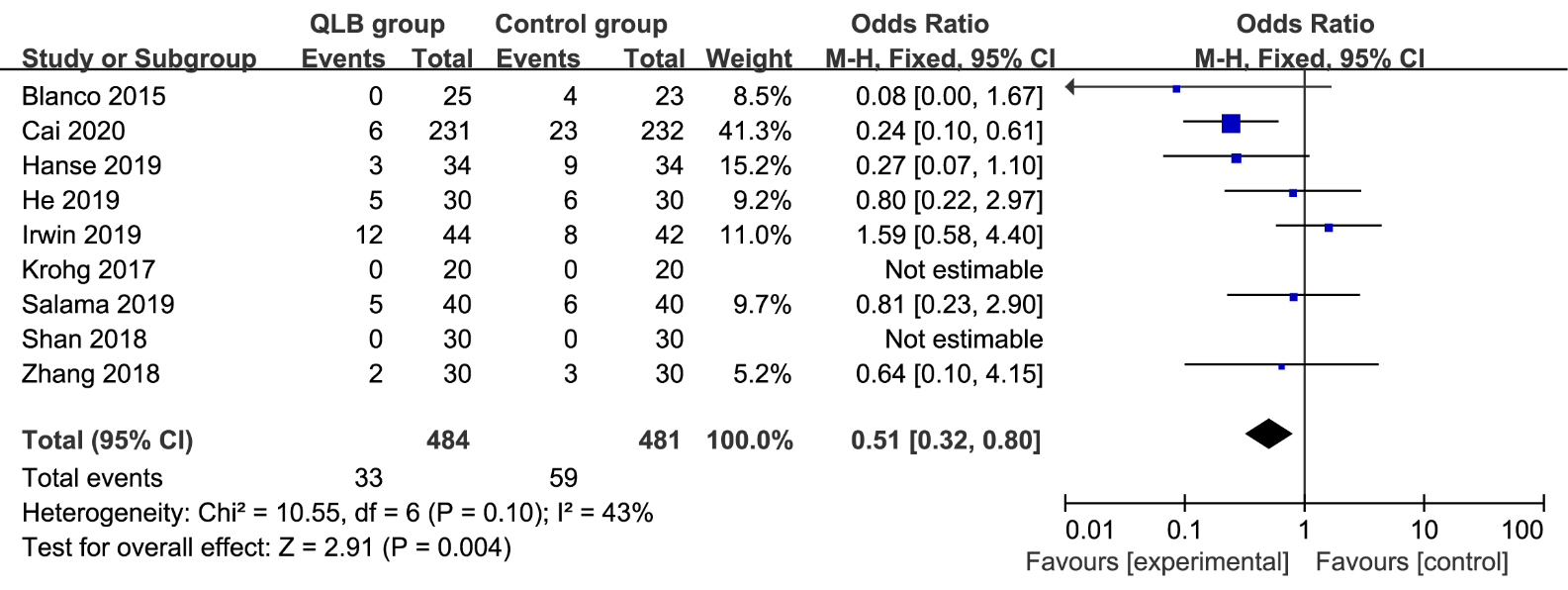
**Supplementary Figure 2.** Forest plot of incidence of postoperative PONV

**Supplementary Figure 3 (Fig 3S).** Funnel plots with Egger’s test for each outcomes.


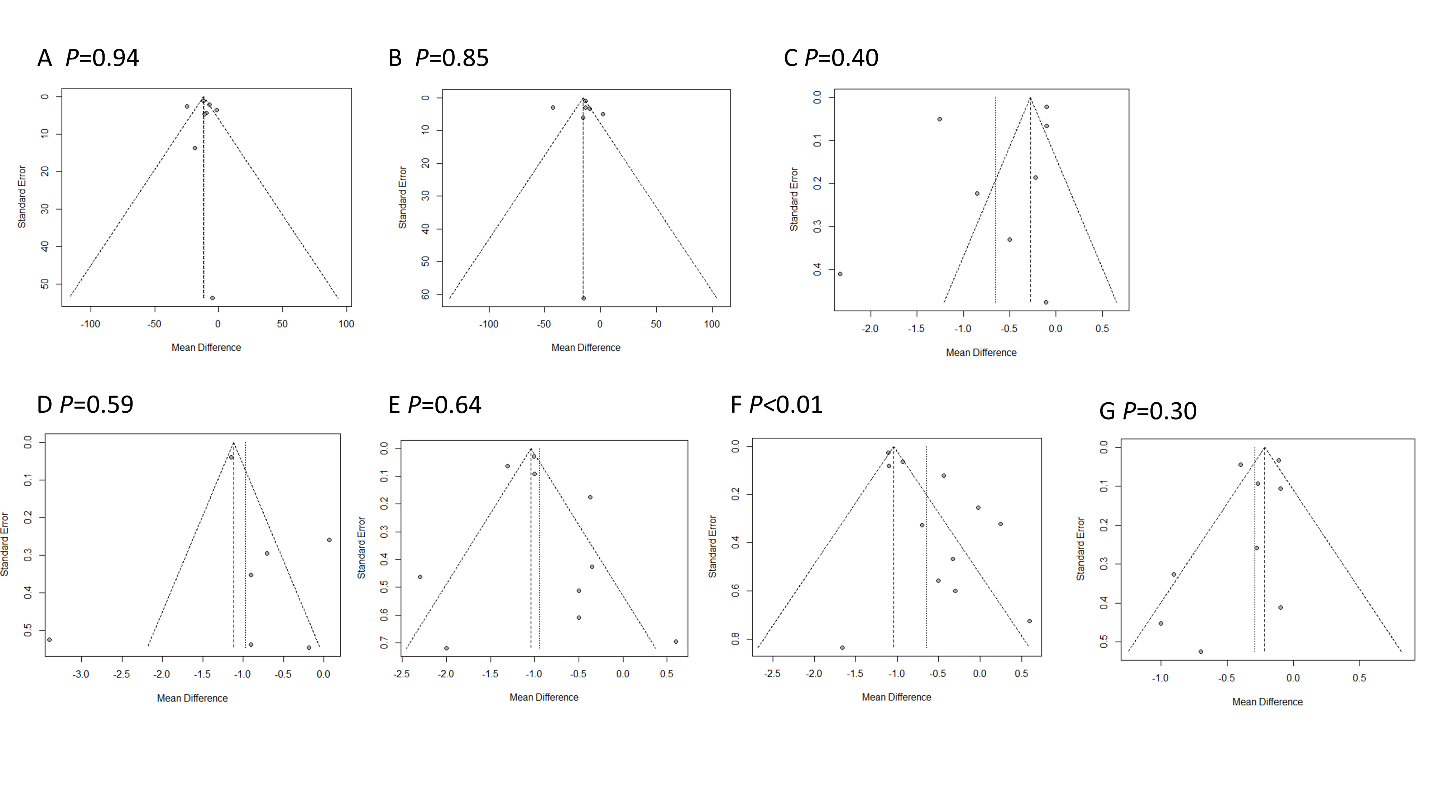


Funnel plots with Egger’s test for cumulative 24-h intravenous morphine equivalent consumption (A), cumulative 48-h intravenous morphine equivalent consumption (B), Pain score at rest 2h postoperatively (C), Pain score at rest 6h postoperatively (D), Pain score at rest 12h postoperatively (E), Pain score at rest 24h postoperatively (F), Pain score at rest 48h postoperatively (G).

**Supplementary Figure 4 (Fig 4S).** Funnel plots with Egger’s test for each outcomes.


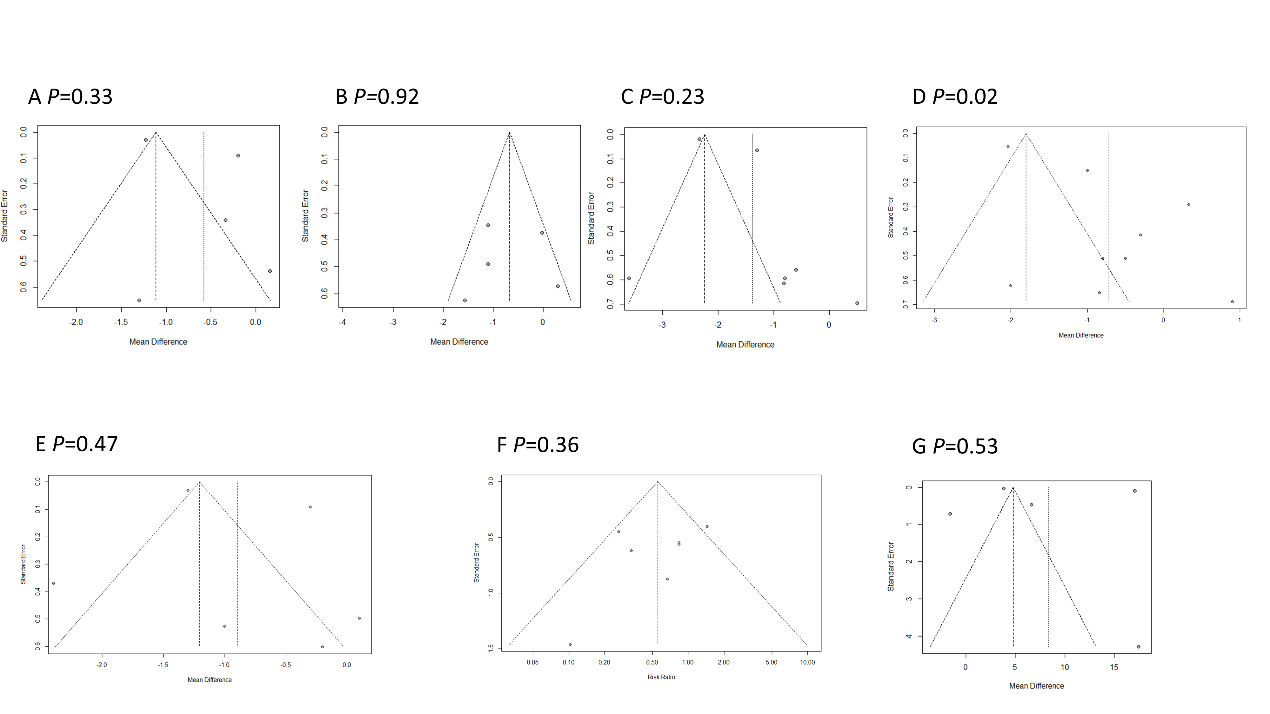


Funnel plots with Egger’s test for pain score at dynamic 2h postoperatively (A), pain score at dynamic 6h postoperatively (B), pain score at dynamic 12h postoperatively (C), pain score at dynamic 24h postoperatively (D), pain score at rest 48h postoperatively (E), incidence of postoperative PONV (F), time to first analgesic request (G).
